# Multi-branch low-light image iterative enhancement network

**DOI:** 10.1038/s41598-025-26962-6

**Published:** 2025-12-03

**Authors:** Yiwen Dou, Yiting Gao, Mei Gao, Senyan Zhao, Chenhao Zeng

**Affiliations:** 1 School of Computing and Information Technology, Anhui Polytechnic University, Wuhu, 241000 China; 2 Key Laboratory of Advanced Perception and Intelligent Control of High-end Equipment, Anhui Polytechnic University, Wuhu, 241000 China

**Keywords:** Engineering, Mathematics and computing, Optics and photonics

## Abstract

Images captured at night or under low-light conditions often suffer from insufficient brightness, low resolution, and detail loss. Although numerous deep learning-based methods have been proposed, most rely on direct mappings from low-illumination to normal-illumination images, which struggle to adapt to diverse real-world conditions. To address these challenges, this paper proposes a Multi-Branch Low-Light Image Iterative Enhancement Network (MBLLIE-Net). Specifically, to enhance feature extraction at different levels, our framework adopts a multi-branch architecture, in which features of various depths and scales extracted by the encoder are processed and refined through multiple parallel branches. To overcome the limitation of insufficient spatial dependency modeling, we introduce a Spatial Recurrent Unit (SRU) within each branch, which effectively captures long-range spatial relationships while preserving local details. Furthermore, to better emphasize salient channels across varying feature dimensions, we propose an Adaptive Receptive Field Channel Attention (ARFCA) module that dynamically adjusts its receptive field according to the channel dimension, enabling precise feature selection with negligible computational overhead. Finally, the decoder fuses the outputs from all branches to generate an initial enhanced result, which is iteratively refined by concatenating it with the original input, ensuring progressive improvement in image quality. Extensive experiments demonstrate that MBLLIE-Net effectively restores illumination, detail, and color fidelity across a wide range of low-light scenarios, outperforming existing single-path approaches in both quantitative metrics and human perceptual evaluations.

## Introduction

Under challenging lighting conditions such as at night or under unstable illumination, captured images often suffer from severely degraded visibility, characterized by low brightness, poor contrast, color distortion, and increased noise^1,2^. These degradations critically impair both human visual perception and the performance of downstream computer vision tasks such as object detection^3,4^ and semantic segmentation^5,6^, making low-light image enhancement (LLIE) a vital research topic.

Early LLIE methods were mainly relied on traditional techniques, including histogram equalization^7–10^, gamma correction^11–13^, and Retinex-based models^14–17^. While they improve visibility to some extent, these approaches often suffer from detail loss, color distortion, and sensitivity to parameter settings, which limits their robustness in diverse real-world scenarios.

The advent of deep learning has significantly advanced LLIE. CNN-based approaches such as LLNet^18^, MBLLEN^19^, and EEMEFN^20^ have demonstrated strong feature extraction and enhancement capabilities, while Retinex-inspired networks like RetinexNet^21^ and MambaLLIE^22^ embed physical priors to improve interpretability and stability. Meanwhile, lightweight frameworks^23–25^ and Transformer-based models^26–28^ further enhance efficiency and long-range dependency modeling. More recent designs explore multi-branch and iterative strategies^29,30^, achieving progressive refinement of illumination and details.

Despite these advancements, existing models still face challenges in faithfully restoring fine-grained details and maintaining color consistency under diverse low-light conditions. Moreover, structural information such as edges and textures in low-light images is often too fragile to be effectively preserved by conventional models at a single scale, which is especially problematic in related critical applications such as transportation. Due to changes in lighting conditions or limitations of imaging equipment, the captured images may have various types of insufficient brightness, and reliable visual perception is crucial for tasks such as traffic monitoring and autonomous driving. Combined with the scarcity of paired training data in real-world environments, these factors restrict the generalization capability of current LLIE models in practical scenarios.

To bridge this gap, we propose MBLLIE-Net. Our key innovations include:

- A novel multi-branch iterative architecture that simplifies complex low-light enhancement by decomposing it into parallel multi-scale feature extraction and progressive fusion, significantly boosting detail recovery.
- The integration of SRU and ARFCA attention within each branch, where SRU enables efficient spatio-temporal feature fusion across scales and ARFCA adaptively highlights critical channels, ensure robust performance under various low-light conditions.
- A joint optimization strategy leveraging strategically weighted pixel loss, multi-scale perceptual loss, adversarial loss, and color loss to effectively address complex degradations like noise, color shift, and detail loss.
- The construction of the TT100K-re paired traffic dataset by simulating low-light conditions on TT100K, addressing the shortage of real-world low-light/normal-light traffic image pairs for training and evaluation.

The remainder of this paper is organized as follows. First, related work is reviewed. Next, the proposed method is elaborated. Then, experimental results and analysis are presented. Finally, conclusions are drawn.

## Related work

### Traditional methods

Traditional methods for enhancing low-light images mainly include histogram equalization, Retinex theory, and gamma correction. Histogram equalization enhances images by exploiting their statistical characteristics to expand the dynamic range of pixel values and thereby improve overall contrast. Specifically, AHE^7^ introduces adaptive histogram equalization by redistributing gray levels within local regions, which enhances local contrast while preserving details. POHE^9^ approximates the local gray-scale distribution with Gaussian parameters and combines the integral graph to quickly estimate the cumulative distribution function (CDF), achieving high real-time local contrast enhancement. Bi-HE^10^ performs histogram partitioning and constrained redistribution to suppress over-enhancement while maintaining overall brightness.

On the other hand, gamma correction adjusts the relative intensity of dark and bright regions to enhance contrast. AGCWD^11^ adaptively determines the gamma value using a probability-weighted distribution, thereby accurately stretching dark regions while suppressing overexposure in bright regions. AGC^12^ dynamically estimates gamma curves based on local statistics to simultaneously enhance contrast and preserve details in a parameter-free manner. FDCIT-GC^13^ combines fuzzy dissimilarity contextual intensity transformation with gamma correction to effectively improve the brightness and contrast of color images.

Unlike direct pixel-value stretching, Retinex-based methods approaches decompose an image into illumination and reflectance components. Retinex theory^14^ originally proposed by Land explains color constancy through such decomposition. NPE^15^ integrates color naturalness constraints into illumination–reflectance estimation, correcting non-uniform lighting while preserving natural color appearance. LIME^17^ rapidly estimates the initial illumination map using structural priors, followed by optimization-based refinement, enabling lightweight yet effective enhancement of low-light images. SRIE^16^ incorporates structural priors and sparse constraints into the Retinex framework, achieving detail-preserving enhancement for low-light images.

Although these methods are effective in enhancing image quality under specific conditions, they still have some limitations when dealing with low light images. Histogram equalization improves image contrast but usually results in a loss of image details. Enhancement methods based on the Retinex theory are designed to simulate the way the human eye perceives light and improve the color reproduction and brightness performance of images, but they are highly sensitive to parameter settings and are prone to color distortion or excessive enhancement of details if not handled properly. Gamma correction, on the other hand, improves image quality by adjusting the luminance profile of the image, but it is more sensitive to the nonuniform distribution of light, and may not be able to equalize the processing of all areas of the image. Thus, while traditional methods remain effective for particular types of low-light images, they are often insufficient for more diverse and challenging scenarios.

### Deep learning-based methods

Deep learning has revolutionized the field of LLIE, offering data-driven solutions that surpass traditional techniques. Early CNN-based methods laid the foundation for applying deep learning to LLIE. Lore et al.^18^ pioneered this direction by designing a deep stacked sparse denoising autoencoder to learn the mapping from low-light to normally lit images directly, demonstrating the potential of deep learning for LLIE. Subsequent research built on this foundation: Ren et al.^31^ designed a hybrid encoder–decoder with spatial recurrent neural networks for boundary details, while Zhu et al.^20^ introduced EEMEFN, which fuses multi-exposure images to reduce noise and color shift.

Another prominent research stream integrates Retinex theory with deep learning, bridging data-driven learning with physical priors. The seminal work RetinexNet^21^ by Wei et al. decomposes an image into illumination and reflectance components through a dedicated decomposition network, followed by an enhancement network for illumination adjustment. Duong et al.^32^ extended Retinex-based modeling by employing multiple specialized networks to collaboratively estimate illumination and reflectance components. Weng et al.^22^ presented MambaLLIE to implicitly embed Retinex-aware priors within a global-then-local state space framework, achieving robust low-light enhancement without explicit image decomposition. More recently, Li et al.^33^ proposed Diff-Retinex++, which integrates Retinex decomposition with diffusion modeling to jointly leverage physical priors and generative restoration capabilities, enabling natural illumination correction and fine-grained detail recovery. These efforts highlight the advantages of embedding physical priors into network design for more interpretable and stable enhancement.

To improve efficiency and stability, lightweight methods have also been proposed. Li et al.^23^ developed LightenNet, which employs a compact CNN to predict the illumination map for brightness correction. Fan et al.^24^ proposed IniRetinex, which improves optimization stability by providing a superior initialization of the illumination map, while Zhang et al.^25^ introduced a self-supervised strategy that automatically generates pseudo–ground truth during training, enabling fast and robust enhancement without paired data.

Beyond CNN architectures, Transformer-based frameworks have gained traction due to their ability to capture long-range dependencies. Wang et al.^26^ proposed LLFormer, which leverages local–global feature interactions to restore details and contrast while suppressing noise. Brateanu et al.^27^ introduced LYT-Net, a dual-path Transformer operating in the YUV color space, which separately models luminance and chroma components through multi-head self-attention to collaboratively enhance brightness and reduce chroma noise. More recently, Yin et al.^28^ presented the Structure-Guided Diffusion Transformer (SDTL), which integrates diffusion modeling with Transformer architecture, leveraging structure-guided attention to preserve spatial consistency and fine textures under extremely low-light conditions.

To further push LLIE performance, more complex architectures have been explored, with multi-branch and iterative strategies emerging as promising directions. Multi-branch frameworks primarily process features across different scales or modalities in parallel to capture rich contextual information, while iterative frameworks focus on progressively refining restoration results via repeated learning loops. Lv et al.^19^ pioneered MBLLEN, an end-to-end multi-branch network integrating feature extraction, enhancement, and fusion. Subsequent works such as MBPNet^29^ and Zeng^34^ further advanced multi-branch architectures through progressive enhancement and hierarchical fusion to preserve fine textures. More recently, Gao et al.^35^ designed a dual-stream modulated network, where one branch focuses on illumination enhancement and the other on super-resolution for the joint restoration of extremely dark images. On the iterative front, Gao et al.^36^ introduced a bi-level adversarial framework that first adjusts global brightness and then refines textures, achieving progressive brightening while suppressing noise amplification. Similarly, Yin et al.^30^ developed ILR-Net, which balances brightness recovery and detail preservation through iterative Retinex-based refinement. Despite these advances, challenges remain in faithfully restoring fine textures and ensuring robustness under diverse real-world conditions. Inspired by the strengths of these two strategies, we integrate a multi-branch structure with an iterative enhancement scheme. Specifically, different branches in our framework integrate local features at varying depths and scales to capture long-range dependencies (addressing texture preservation), while the iterative scheme progressively improves overall image quality by refining illumination and details in loops (enhancing robustness).

## Proposed method

**Fig. 1:**
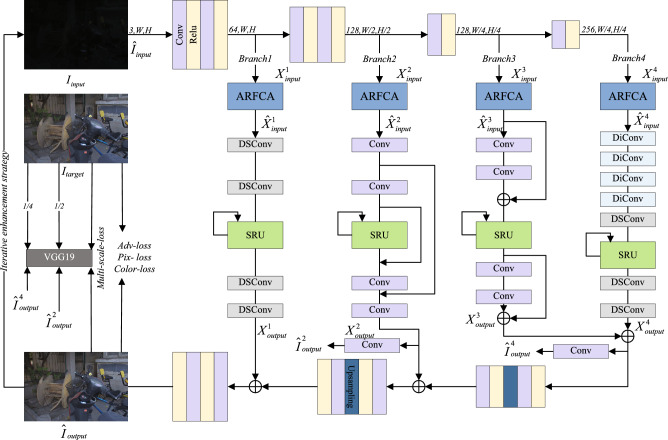
Overview of the proposed MBLLIE-Net.

In this section, we detail the specific architecture of the proposed MBLLIE-Net. Its overall architecture diagram is presented in Fig. 1.

### Overall network architecture

The proposed MBLLIE-Net adopts an overall U-Net–like structure, replacing the traditional skip connections with a multi-branch enhancement structure in the middle. The encoder processes the input images to extract features at four different depths and scales. The multi-branch enhancement network enhances and fuses these multi-depth and multi-scale features. The decoder then receives the enhanced features from the multi-branch network to perform feature fusion and reconstruction, generating generating a preliminary enhancement result. Finally, through an iterative enhancement strategy, the current enhanced output is first concatenated along the channel dimension with the original input image, and this concatenated result is then repeatedly fed back into the encoder. This iterative process undergoes multiple iterations to produce the final enhanced result.

The encoder consists of four convolutional blocks. Each block contains one or two convolutional layers, after each convolutional layer, the ReLU activation function is applied, aiming to enhance the model’s nonlinear fitting capability. The formula for the encoder part is given below:

1 $$\begin{aligned} X_{input}^i=f_{encoder}^i\left( \widehat{I}_{input}\right) ,i=1,2,3,4,\end{aligned}$$

where $\widehat{I}_{input}$ is the input to the encoder and $f_{encoder}^i$ is the feature extractor for the i-th convolutional block within the encoder.

The decoder structure is similar to the encoder, consisting of three convolutional blocks, each containing two convolutional layers and two ReLU activation functions, and the first two convolutional blocks also include an upsampling operation.

### ARFCA

**Fig. 2:**
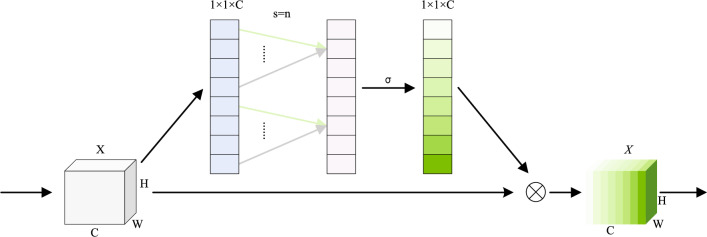
Architecture of the Adaptive Receptive Field Channel Attention (ARFCA) module.

Channel attention has proven effective in many image restoration tasks by emphasizing informative feature channels while suppressing less useful ones. To better meet the requirements of low-light image enhancement, we propose an Adaptive Receptive Field Channel Attention (ARFCA) module. Unlike conventional channel attention mechanisms with a fixed kernel size, and unlike efficient variants such as ECA (which uses lightweight log-guided scaling) and SK (which performs multi-branch fusion), ARFCA defines the 1D kernel size as a logarithmic function of the channel dimension with odd-kernel enforcement and range clipping, enabling stable, smoothly scaled receptive-field adaptation with negligible overhead. The overall architecture is illustrated in Fig. 2.

Concretely, given an input feature map $X \in \mathbb {R}^{B\times C\times H\times W}$, ARFCA first applies global average pooling, denoted as $G(\cdot )$, to aggregate spatial information and obtain a compact channel descriptor $G(X) \in \mathbb {R}^{B\times C\times 1\times 1}$. Next, a 1D convolution is applied along the channel dimension with an adaptively determined kernel size *s*:

2 $$\begin{aligned} s=\min \!\left\{ \,s_{\max },\;\max \!\left\{ \,s_{\min },\;2\left\lfloor \tfrac{1}{2}\,\operatorname {round}\!\bigl (\tfrac{\log _{\beta } C}{\gamma }\bigr )\right\rfloor +1\right\} \right\} . \end{aligned}$$

where $\operatorname {round}(\cdot )$ rounds to the nearest integer, $\beta$ and $\gamma$ are hyper-parameters, and $s_{\min },s_{\max }\in (2\mathbb {N}+1)$ bound the kernel size.

The channel weights are generated by feeding the convolved descriptor into a Sigmoid function:

$$\omega =\sigma \!\bigl (\textrm{Conv}^{\,s}_{1\textrm{D}}(G(X))\bigr ),$$

where $\textrm{Conv}^{\,s}_{1\textrm{D}}(\cdot )$ denotes a 1D convolution with kernel size *s*. Finally, the input is rescaled channel-wise as $Y=X\odot \omega$ to enhance informative channels and suppress redundant ones. Unless otherwise specified, we set $\beta =3$, $\gamma =1$, $s_{\min }=3$, and $s_{\max }=9$.

### SRU

**Fig. 3:**
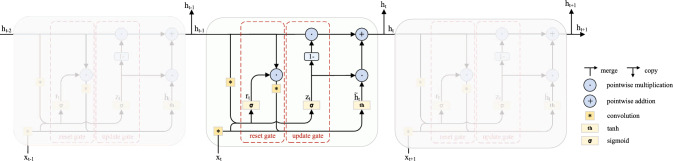
Spatial Recurrent Unit.

In iterative enhancement, repeated refinement can gradually amplify noise and artifacts, leading to overfitting. Traditional Recurrent Neural Networks (RNNs)^37^ also suffer from vanishing and exploding gradients, making long-range dependencies difficult to capture in practice. To address these limitations while enhancing spatial modeling capability, and inspired by the Gated Recurrent Unit (GRU)^38^, we introduce a Spatial Recurrent Unit (SRU). While retaining the temporal modeling advantages of gated units, SRU strengthens spatial feature extraction by combining dilated-convolutional gating with a depthwise–pointwise separable candidate path and an adaptive state-alignment mechanism. The overall structure is illustrated in Fig. 3.

Concretely, the SRU operates on intermediate feature maps rather than raw images. We add the SRU module to each enhanced branch. At each iteration *t*, it receives the current feature map $x_t \in \mathbb {R}^{B \times C \times H \times W}$ and the previous hidden state $h_{t-1}$ of this stage, and outputs $h_t$. If the spatial resolutions differ, $h_{t-1}$ is bilinearly resampled to match $x_t$ before concatenation, so that SRU can progressively refine spatio-temporal representations across iterations.The process is calculate as follows:

3 $$\begin{aligned} z_t=\sigma \left( {C_d}([h_{t-1},x_t];W_z)\right) \end{aligned}$$

4 $$\begin{aligned} r_t=\sigma \left( {C_d}([h_{t-1},x_t];W_r)\right) \end{aligned}$$

5 $$\begin{aligned} \tilde{h}_t=\tanh \left( C_{sep}([r_t\odot h_{t-1},x_t];W_h)\right) \end{aligned}$$

6 $$\begin{aligned} h_t=(1-z_t)\odot h_{t-1}+z_t\odot \tilde{h}_t\end{aligned}$$

where $z_t$ and $r_t$ are the update and reset gates; $\tilde{h}_t$ and $h_t$ denote the candidate and current hidden states; $x_t$ is the current input feature map, and $W_i$ represents the weight parameters of the update gate, the reset gate, and the candidate state; $[\cdot ,\cdot ]$ denotes channel concatenation, $\odot$ denotes element-wise multiplication, $\sigma$ is the sigmoid function, and $\tanh$ is the hyperbolic tangent function. $C_d$ and $C_{sep}$ respectively represent the dilated convolution operation and the depthwise-separable convolution operation. Operating on feature maps with gated residual updates regularizes the iterative refinement and mitigates overfitting, while maintaining spatial detail and computational efficiency.

### Multi-branch enhanced network

This paper designs four distinct enhancement branches to learn features at different scales, with each branch tailored to the characteristics of its corresponding feature level.

Specifically, the first branch adopts four depthwise-separable convolutions. Shallow features are mainly composed of low-level details such as textures and edges, which can be effectively captured without complex operations. Depthwise separable convolutions are particularly suitable for this scenario, as they significantly reduce the number of parameters and computational cost while still preserving and enhancing fine-grained local details.

The second branch employs four standard convolutional layers enhanced by skip connections. Mid-level features contain both structural information and local semantics, requiring a stronger representational capacity than shallow layers. Standard convolutions can capture this complexity, while skip connections ensure smooth information flow across layers, preventing the loss of mid-level structural cues during deeper propagation.

The third branch introduces two residual blocks. As the network goes deeper, feature representations become increasingly abstract and semantic patterns grow in complexity. In this context, residual learning offers distinct advantages: it establishes a direct information pathway between input and output, stabilizing the optimization process and effectively alleviating gradient vanishing or explosion problems commonly encountered during deep network training.

The fourth branch utilizes depthwise separable convolutions combined with dilated convolutions. Deepest features are highly semantic and demand broad contextual awareness to be fully exploited. Dilated convolutions expand the receptive field and capture long-range dependencies without a significant increase in computational cost, while depthwise separable convolutions maintain efficiency. This combination balances expressiveness and efficiency, ensuring that global context is modeled effectively while keeping the computational burden under control. These four branches can be expressed as:

7 $$\begin{aligned} X_{output}^i=f_{Branch}^i(\widehat{X}_{input}^i),i=1,2,3,4,\end{aligned}$$

where $f_{Branch}^i$ denotes the $i$-th branch. The fourth branch has an output channel count that is half of its input channel count, while for all other branches, the input and output channel counts remain identical.

For efficient fusion of multi-branch features, the enhanced feature maps outputted from the third and fourth branches are first summed element-wise and the size of the feature map is doubled through upsampling. Subsequently, this merged feature is added to the enhanced feature map outputted from the second branch on an element-by-element basis, and the feature map size is then tripled through upsampling. Finally, the result is summed with the low-level feature map output from the first branch, and the number of channels is compressed to three (RGB) using a $1\times 1$ convolutional layer to match the spatial dimensions and color space of the target image. The above operations are expressed as:

8 $$\begin{aligned} \widehat{I}_{output} = F_{gz}\left( X_{output}^1 + F_{up}\left( X_{output}^2 + F_{up}\left( X_{output}^3 + X_{output}^4 \right) \right) \right) \end{aligned}$$

where, $F_{up}$ indicates the up-sampling operation, and $F_{gz}$ indicates the operation of compressing the fused feature map to the RGB channel.

In addition, each branch of the multi-branch augmentation network includes an ARFCA channel attention module at its front part, which is represented as:

9 $$\begin{aligned} \widehat{X}_{input}^i=X_{input}^i\sigma \left( Conv_{1D}^k\left( GAP\left( X_{input}^i\right) \right) \right) ,\end{aligned}$$,

where, $X_{input}^i$ represents the original input feature, $\widehat{X}_{input}^i$ represents the generated feature, $\text {GAP}$ represents the average pooling operation of the input feature,then the weights of each channel are calculated by the convolution operation $Conv_{1D}^k$ and the sigmoid activation function. Finally, an element-wise multiplication is performed between these weights and the original input feature, resulting in the production of the final output feature map.

Furthermore, each branch incorporates an SRU. The core of the SRU lies in its gated mechanism (including an update gate and a reset gate), which selectively retains or forgets prior information. By controlling the flow of information, it helps prevent the overfitting of the model. In our network, the SRU receives the hidden state from the previous iteration in the same stage and the feature map of the current iteration, and performs gated updates on these features, which not only suppresses iterative overfitting but also steadily improves brightness and detail.

### Iterative enhancement strategy

**Fig. 4:**
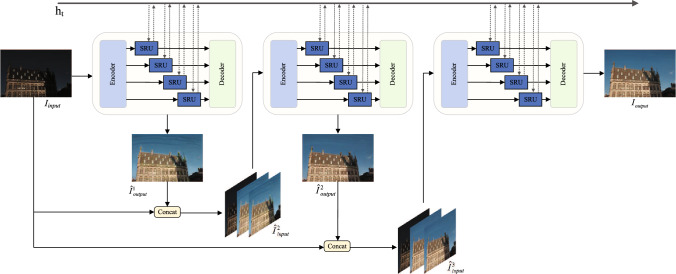
Iterative enhancement strategy.

The proposed MBLLIE-Net employs an iterative enhancement strategy to improve model performance. We empirically determined that three iterations yield optimal results, as elaborated in the ablation study section. We concatenate the two enhanced outputs with the original image along the channel dimension to generate new inputs for the encoder. This approach serves a dual purpose: preserving the original image’s characteristics helps prevent feature degradation, while the repeated integration of enhanced images directs the network’s attention to the improved features. The mathematical representation of this process is as follows:

10 $$\begin{aligned} \widehat{I}_{input}^n=Concat\left\{ I_{input},\widehat{I}_{output}^{n-1},\widehat{I}_{output}^{n-1}\right\} ,n=2,3,\end{aligned}$$

where, $\widehat{I}_{output}^{n-1}$ is the output before the n-th iteration, and the original image $I_{input}$ is concatenated to obtain $\widehat{I}_{input}^n$, which serves as the input for the n-th iteration. It is worth noting that in the first round of iterative enhancement, the encoder’s initial input $\widehat{I}_{input}^n$ is the original low-light image $I_{input}$. The specific unfolded structure of the iterative enhancement network is presented in Fig. 4.

### Loss function

To enhance the visual quality of the generated images, we employ four loss functions to oversee the training process: adversarial loss, pixel loss, color loss and multi-scale loss.

#### Adversarial loss

Adversarial loss is a frequently used loss function in deep learning, especially in GANs. It is the core mechanism of GAN training and optimization, which promotes continuous competition and improvement between the generator and the discriminator. In this paper, the model as a whole is used as the generator $G(I_L;\theta )$, and discriminator $\textrm{D}$ is used to distinguish between real and generated images. Its adversarial loss function can be expressed as:

11 $$\begin{aligned} L_D=L_{MSE}(D(G(I_L),0)+L_{MSE}(D(I_H),1),\end{aligned}$$

where $D(G(I_{_L}),0)$ denotes the probability that the generated image $G(I_L)$ is judged as false and $(D(I_H),1)$ denotes the probability that the ground truth $I_{H}$ will be judged as true. In addition, the mean square error loss function $L_{MSE}$ is utilized to measure the disparity between the actual and desired outputs. The adversarial loss function of the generator is given as:

12 $$\begin{aligned} L_{Ad\nu }=L_{MSE}(D(G(I_L)),1).\end{aligned}$$

#### Pixel loss

We apply MSE to measure the likeness between the generated image and the ground truth image. In particular, as our iterative refinement process progresses, we calculate the MSE for each iteration between the generated image and the ground truth. These results are then weighted and accumulated. The formula is as follows:

13 $$\begin{aligned} L_{Pixel}=\sum _{i=1}^Nc^{N-i}L_{MSE}(\widehat{I}_H^i,I_H),\end{aligned}$$

where, $\widehat{I}_i$ represents the generated image, $I_{H}$ denotes the ground truth, $\text {N}$ represents the iteration count, $L_{MSE}$ signifies the MSE calculation between the generated and ground truth, and c we empirically set to 0.8.

#### Color loss

In low-light image enhancement, the colors of the generated images are often prone to deviation, leading to color distortion problems. Although pairs of image data are used in the training process, significant color style differences still exist between different datasets. To greatly enhance the natural color accuracy of the enhanced image, this paper introduces a color loss function, which is expressed as:

14 $$\begin{aligned} L_{Color}=\sum _qsim((\widehat{I}_H)_q,(I_H)_q),\end{aligned}$$

where $sim(,)$ represents the cosine similarity of pixel-level color vectors between the generated and the ground truth, and $\text {q}$ denotes the index of the qth pixel in the generated or ground truth.

#### Multi-scale perceptual loss

The perceptual loss function enhances the model’s ability to comprehend and depict the content of images by analyzing their high-level features,which can effectively maintain the texture and intricate details of the images, leading to an improvement in overall image quality. In this paper, we employ a pre-trained VGG19 network to extract multi-scale perceptual features from both the generated and ground truth, and the evaluation process involves computing the mean square error between these two sets of features. By using three different scale branches in the network to formulate a multi-scale perceptual loss, the formula is as follows:

15 $$\begin{aligned} L_{Multi}=L_{VGG}(\widehat{I}_{output},I_{target})+\gamma _{2}L_{VGG}(\widehat{I}_{output}^{2},I_{target}^{2})+\gamma _{4}L_{VGG}(\widehat{I}_{output}^{4},I_{target}^{4}), \end{aligned}$$

where $L_{VGG}(\cdot ,\cdot )$ denotes the disparity between the generated image and the real one measured by comparing the features extracted by the VGG19 network at a particular layer. Essentially, a smaller $L_{VGG}(\cdot ,\cdot )$ indicates that the generated image bears a closer resemblance to the actual image. $I_{targ et}^i$ and $\widehat{I}_{output}^i$ denote the ground truth and the enhanced image with a scale size i times that of the original image, respectively. The trade-off constants $\gamma _{2}$ and $\gamma _{4}$ are manually set to 0.8 and 0.4.

#### Overall loss

Finally, the overall objective function is formulated as a weighted combination of the above loss terms:

16 $$\begin{aligned} L_{total} = \lambda _{1} L_{Adv} + \lambda _{2} L_{Pixel} + \lambda _{3} L_{Color} + \lambda _{4} L_{Multi}, \end{aligned}$$

where $\lambda _{1}, \lambda _{2}, \lambda _{3}, \lambda _{4}$ are trade-off parameters that balance the contributions of adversarial, pixel, color, and perceptual supervision. In our experiments, we set $\lambda _{1}=0.01$, $\lambda _{2}=1.5$, $\lambda _{3}=0.5$, and $\lambda _{4}=1$, based on validation performance, which yielded satisfactory results. The choice of these coefficients was guided by validation performance: a relatively larger weight on pixel loss helps preserve structural fidelity, while a smaller adversarial weight mitigates training instability. Ultimately, this configuration strikes a good balance among fidelity, realism, and color accuracy, making the model more adaptable to image enhancement under varying lighting conditions.

## Experimental results and analysis

The computer environment used in our experiment is as follows: The CPU configuration is Intel Core i9-10900X, the GPU is RTX 4090, the system memory is 64GB, the operating system is Ubuntu 22.04, the software used for training and testing is Visual Studio Code, the Python version is 3.9, and the Pytorch version is 2.0.1. All neural networks are trained, validated, and tested on the PyTorch deep learning framework, which supports GPU acceleration to significantly reduce training time.

### Datasets and metrics

We conducted experiments on multiple benchmark datasets, including LOL-v1^21^, LOL-v2^39^, TT100K-re, DICM, LIME, and NPE. Among these, the LOL-v1 dataset contains 500 pairs of normal-light and low-light images sourced from real-world scenes. Out of these, 485 pairs are used for training, while 15 pairs are reserved for testing. The LOL-v2 dataset consists of both real-captured and synthetic image pairs. We choose the synthetic image pairs as our training data and denote them as LOLv2-Synthetic, with 900 pairs used for training and 100 pairs for testing.To evaluate performance in practical traffic scenarios, we constructed the TT100K-re dataset based on TT100K^40^. Specifically, 500 pairs of traffic-scene images were generated, where normal-light images were extracted from TT100K, and their corresponding low-light counterparts were synthesized by adjusting gamma and resolution. Following the same split as LOL-v1, 485 pairs are used for training and 15 pairs for testing.

For quantitative analysis, we adopt four widely used evaluation criteria: PSNR (Peak Signal-to-Noise Ratio), SSIM (Structural Similarity Index Measure), MSE (Mean Squared Error), and LPIPS (Learned Perceptual Image Patch Similarity). In addition, since the DICM, LIME, and NPE do not provide ground-truth images, we further employ three no-reference image quality metrics, namely NIQE (Natural Image Quality Evaluator), BRISQUE (Blind/Referenceless Image Spatial Quality Evaluator), and PIQE (Perception-based Image Quality Evaluator)-to assess visual quality on these real-world datasets.

### Training details

To enhance computational efficiency during training, we resize the images to $256\times 256$, while the test images retain their original sizes. For optimization, we employ the Adam optimizer and use its default parameter settings. Batch size is set to 2, and the initial learning rate is $3\times 10^{-4}$, which is then decayed to $1.5\times 10^{-4}$. To ensure stable convergence across datasets, we set the number of training epochs to 500.

### Evaluation and comparison

Our MBLLIE-Net was benchmarked against nine existing low-light image enhancement techniques, including KinD^41^, DeepUPE^42^, KinD++^43^, RetinexNet^21^, MBLLEN^19^, PairLIE^44^, SHAL-Net^45^, MSRNet^46^, MBPNet^29^.

**Table 1:** Comparison of average PSNR/SSIM/MSE/LPIPS on the LOL-v1, LOLv2-Synthetic and TT100K-re test datasets.

Method	LOL-v1				LOLv2-Synthetic				TT100K-re			
	PSNR$\uparrow$	SSIM$\uparrow$	MSE$\downarrow$	LPIPS$\downarrow$	PSNR$\uparrow$	SSIM$\uparrow$	MSE$\downarrow$	LPIPS$\downarrow$	PSNR$\uparrow$	SSIM$\uparrow$	MSE$\downarrow$	LPIPS$\downarrow$
KinD^41^	16.66	0.736	102.019	0.270	19.17	0.816	92.348	0.223	17.22	0.802	100.470	0.256
DeepUPE^42^	17.28	0.507	99.605	0.357	19.05	0.838	94.587	0.157	21.00	0.841	80.160	0.158
KinD++^43^	17.67	0.786	100.197	0.232	17.67	0.786	100.197	0.232	19.95	0.754	99.790	0.223
RetinexNet^21^	17.72	0.709	104.587	0.391	19.2	0.800	98.784	0.248	25.78	0.861	41.342	0.188
MBLLEN^19^	17.90	0.762	121.297	0.159	18.62	0.765	99.452	0.287	21.96	0.900	105.614	0.201
PairLIE^44^	18.55	0.743	103.833	0.243	19.25	0.794	96.256	0.230	22.91	0.842	90.212	0.195
SHAL-Net^45^	20.47	0.791	95.436	0.209	18.71	0.799	96.516	0.243	18.37	0.805	107.396	0.214
MSRNet^46^	21.69	**0.845**	90.501	0.144	24.06	0.927	80.854	0.056	28.42	0.957	33.789	0.047
MBPNet^29^	*22.83*	0.825	*88.323*	*0.132*	*25.29*	*0.923*	*73.699*	*0.065*	*32.56*	*0.955*	*22.557*	*0.040*
**Ours**	**23.33**	*0.829*	**83.627**	**0.116**	**25.48**	**0.931**	**72.879**	**0.055**	**33.50**	**0.957**	**19.452**	**0.033**

The best results are marked with bold, and the second-best results are marked with italics.

#### Quantitative assessment

Table1 confirms the significant advantages of our method on the LOL-v1, LOLv2-Synthetic, and TT100K-re datasets.

On the LOL-v1 dataset, our method outperforms competitors in PSNR, MSE, and LPIPS, ranking second in SSIM. Specifically, its PSNR reaches 23.33 dB (2.2% higher than the second best), indicating a better signal-to-noise ratio and improved detail preservation in the enhanced images. The MSE is 83.627 (5.3% lower than the second best), highlighting superior pixel-level enhancement accuracy. The LPIPS value drops to 0.116 (12.1% lower than the second best), reflecting minimized perceptual differences between enhanced and ground truth. While SSIM improvement is modest (0.5% higher than the second-best, 0.829), it still confirms the overall superiority of our method.

Similar trends are observed on the LOLv2-Synthetic dataset, where our method outperforms all competitors across all metrics. Most notably, its LPIPS is 15.4% lower than that of the second best–the largest reduction–indicating robust preservation of texture and structural details. Other improvements include a 1.1% lower MSE, 0.9% higher SSIM, and 0.7% higher PSNR, collectively validating the model’s adaptability to scenes with uneven illumination.

The TT100K-re dataset further confirms the superiority of our method, with our method achieving the best performance across all metrics. Compared with the second best , PSNR and SSIM increase by 2.9% and 0.2%, respectively. For error-related metrics, MSE is reduced by 13.8%, while LPIPS decreases by 17.5%, demonstrating the model’s stability in high-precision enhancement tasks.

#### Qualitative evaluation

**Fig. 5:**
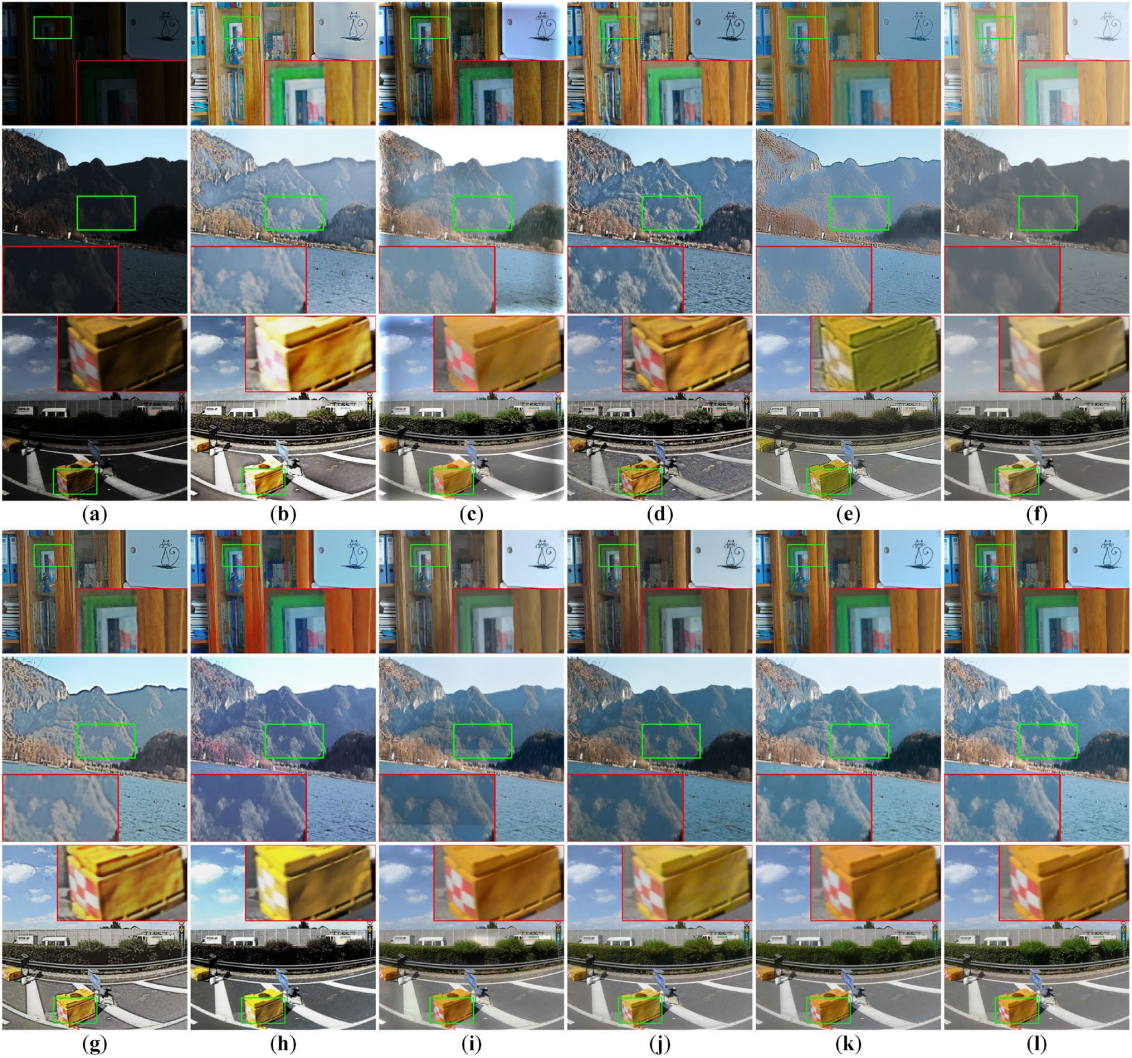
Enhancement comparison on LOL-v1, LOL-v2-Synthetic and TT100K-re. (**a**) Input. (**b**) KinD. (**c**) DeepUPE. (**d**) KinD++. (**e**) RetinexNet. (**f**) MBLLEN. (**g**) PairLIE. (**h**) SHAL-Net. (**i**) MSRNet. (**j**) MBPNet. (**k**) Ours. (**l**) GT.

To further highlight the superior performance of our method, we select representative results for a detailed visual comparison, with Fig. 5 presenting outcomes on the LOL-v1, LOLv2-Synthetic, and TT100K-re datasets.

**Fig. 6:**
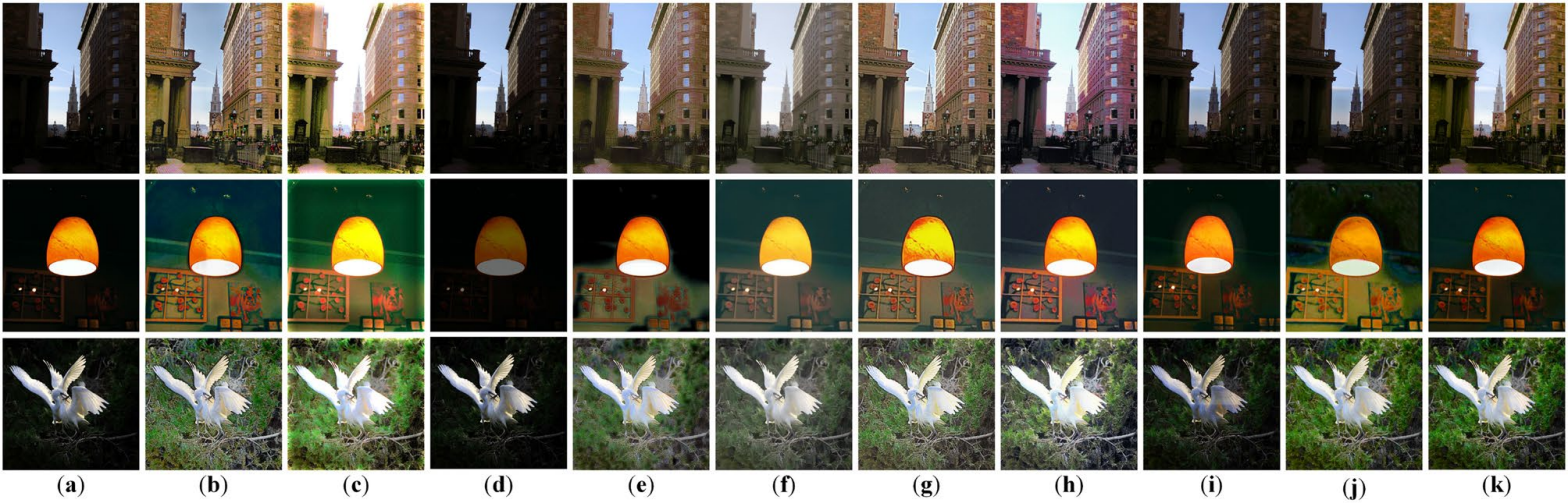
Enhancement comparison on DICM, LIME and NPE. (**a**) Input. (**b**) KinD. (**c**) DeepUPE. (**d**) KinD++. (**e**) RetinexNet. (**f**) MBLLEN. (**g**) PairLIE. (**h**) SHAL-Net. (**i**) MSRNet. (**j**) MBPNet. (**k**) Ours.

Visually, the images enhanced by our method are closer to the ground truth, featuring clearer details and more natural, vivid colors. Specifically, on the LOL-v1 dataset, our method accurately restores the colors of the bookshelf and the books inside, achieving a close match with the ground truth, whereas the results of other methods are generally either too light or too dark–in particular, SHAL-Net produces an obvious reddish distortion on the bookshelf. On the LOLv2-Synthetic dataset, our method effectively recovers the mountain colors in the landscape image, aligning them with the ground truth. In contrast, DeepUPE introduces noticeable white borders, MSRNet exhibits color banding, MBPNet produces overall darker results, while RetinexNet and MBLLEN suffer from detail loss.On the TT100K-re dataset, KinD leads to overexposure; RetinexNet enhances the box to a yellow-green tone; MBLLEN produces a pale yellow result; and KinD++, PairLIE, and SHAL-Net show insufficient enhancement on the sides of the box, leaving deep shadows. MSRNet achieves colors closer to the real ground but introduces additional glare on the background wall. Overall, our method consistently generates visually natural and detail-preserving results that closely match the ground truth across different datasets, further demonstrating strong generalization and robustness in diverse low-light scenarios.

#### Evaluation on real-world images

To further examine the generalization ability of our method on real-world images, we conducted evaluations on three widely used benchmarks, namely DICM, LIME, and NPE. In this experiment, we directly applied the model trained on the LOLv2-Synthetic dataset without any fine-tuning. Since LOLv2-Synthetic covers diverse degradations and scene variations, it provides a representative source model for assessing robustness under different real-world low-light conditions. The visualization results of the DICM, LIME, and NPE datasets are shown in Fig. 6. It can be seen that different methods exhibit certain limitations in low-light image enhancement. Specifically, KinD++ and MSRNet show limited effectiveness in brightness improvement, while DeepUPE often leads to overexposure. The results of KinD, RetinexNet, SHAL-Net, and MBPNet still suffer from a lack of naturalness, and MSRNet further introduces noticeable artifacts. In contrast, our method enhances brightness while preserving fine details and achieves more natural visual results.

Table 2 summarizes the average no-reference metric scores of different methods. Across the nine evaluation results on three datasets, our method achieves the best performance on four metrics and ranks second on another four. Although the BRISQUE score on the LIME dataset is not the best, the visual results still demonstrate satisfactory naturalness and effective detail preservation.

**Table 2:** Comparison of average NIQE/BRISUQE/PIQE on the DICM,LIME and NPE test datasets.

	DICM			LIME			NPE		
Method	NIQE$\downarrow$	BRISUQE$\downarrow$	PIQE$\downarrow$	NIQE$\downarrow$	BRISUQE$\downarrow$	PIQE$\downarrow$	NIQE$\downarrow$	BRISUQE$\downarrow$	PIQE$\downarrow$
Kind^41^	3.084	25.539	33.766	3.723	29.935	33.802	3.448	26.394	37.674
DeepUPE^42^	3.275	25.399	33.341	3.472	**19.757**	34.683	3.810	26.976	37.303
Kind++^43^	8.616	29.728	43.567	6.127	33.731	44.646	8.342	36.275	38.759
RetinexNet^21^	4.775	23.457	25.876	5.815	28.306	*32.677*	3.995	27.888	*35.554*
MBLLEN^19^	2.886	34.365	46.752	3.663	35.074	46.305	3.798	34.974	50.039
PairLIE^44^	3.158	33.293	32.795	4.034	30.409	36.116	3.478	24.966	44.565
SHAL-Net^45^	2.880	31.633	37.667	3.660	31.737	42.242	4.078	33.559	48.352
MSRNet^46^	2.855	20.192	*24.887*	**3.318**	*25.157*	33.541	3.235	23.748	37.295
MBPNet^29^	*2.646*	**19.577**	25.078	3.485	26.532	32.702	*3.163*	**23.026**	37.550
**Ours**	**2.629**	*19.660*	**24.825**	*3.444*	26.867	**32.165**	**3.119**	*23.286*	**32.844**

### Ablation study

In this subsection, we conduct ablation studies to illustrate the impact of the essential components in the proposed MBLLIE-Net, including the multi-branch structure, the SRU module, the ARFCA module, the iterative enhancement strategy, and the loss function.

#### Multi-branch structure

To analyze the contribution of each enhancement branch operating at different scales within the multi-branch enhancement network, we conducted a series of ablation experiments. Specifically, by gradually adding Branch 4, Branch 3, Branch 2, and Branch 1 to the network, we verified the effectiveness of each branch and demonstrated the overall benefit of the multi-branch design.

**Fig. 7:**
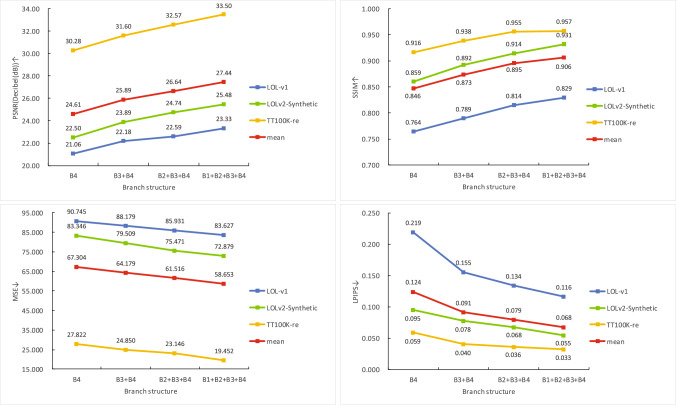
Evaluation of average PSNR, SSIM, MSE and LPIPS values for branch structure variations on three test datasets.

**Fig. 8:**
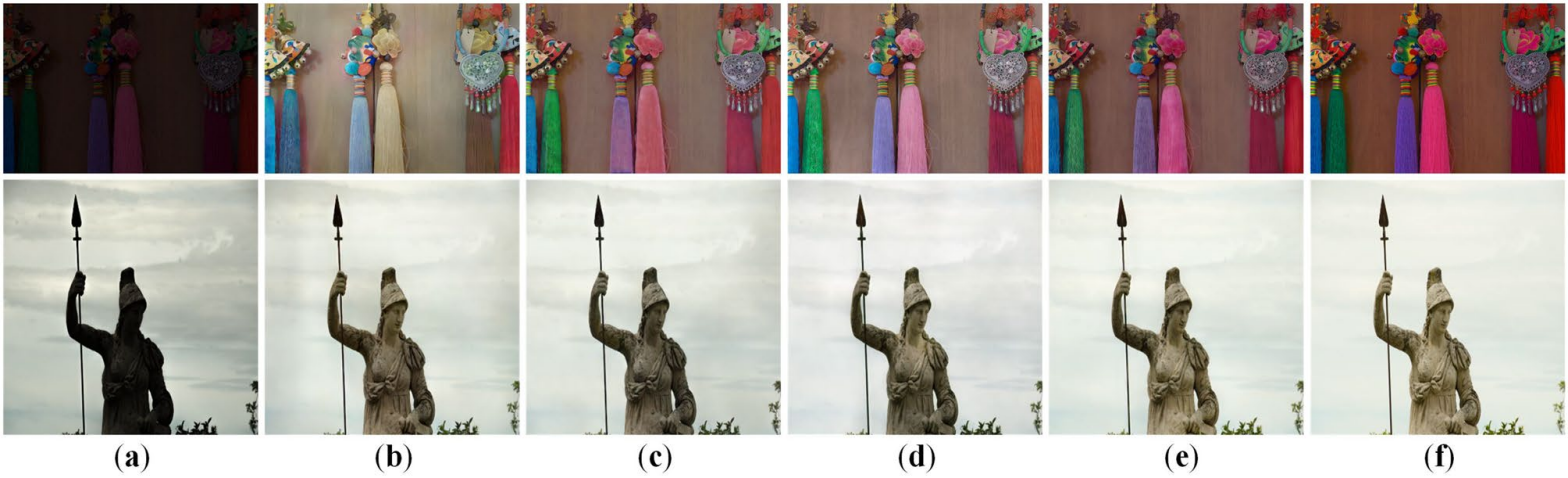
Visual comparison of different branch structures. (**a**) Input. (**b**) B4. (**c**) B3+B4. (**d**) B2+B3+B4. (**e**) B1+B2+B3+B4 (Ours). (**f**) GT.

In addition, we conducted an evaluation on three test datasets, analyzing the statistical trends of the average PSNR and SSIM scores as the branch structure changed. As depicted in Fig. 7, for all three test datasets, both PSNR and SSIM scores steadily improve with an increase in the number of branches. Specifically, the PSNR value rises from a baseline of 24.61 to 27.44 (a relative gain of 11.50%), while the SSIM score improves from 0.846 to 0.906 (a 7.09% relative increase), indicating significant enhancements in pixel-level fidelity and structural similarity.Concurrently, the error metrics demonstrate consistent declines: the MSE value decreases sharply from 83.346 to 58.653 (a 12.85% reduction), and the LPIPS score drops from 0.124 to 0.068 (a 45.16% improvement), suggesting that additional branches effectively enhance low-light image quality.Visual comparisons of different branch configurations in Fig. 8 further reveal that images processed with more branches exhibit progressively closer brightness and richer colors compared to normal-lit scenes. However, increasing the number of branches also incurs higher computational costs. To balance resource efficiency and runtime performance, our network ultimately adopts four branches as the optimal configuration.

**Table 3:** Evaluation of PSNR and SSIM for ablation on SRU, ARFCA across three test datasets.

Model	LOL-v1				LOLv2-Synthetic				TT100K-re			
	PSNR$\uparrow$	SSIM$\uparrow$	MSE$\downarrow$	LPIPS$\downarrow$	PSNR$\uparrow$	SSIM$\uparrow$	MSE$\downarrow$	LPIPS$\downarrow$	PSNR$\uparrow$	SSIM$\uparrow$	MSE$\downarrow$	LPIPS$\downarrow$
w/o SRU	20.46	0.815	95.158	0.135	22.70	0.915	84.410	0.073	32.33	0.956	22.173	0.041
w/o ARFCA	23.15	0.824	84.821	0.140	24.54	0.927	79.559	0.061	32.52	0.954	21.620	0.040
**Ours**	**23.33**	**0.829**	**83.627**	**0.116**	**25.48**	**0.931**	**72.879**	**0.055**	**33.50**	**0.957**	**19.452**	**0.033**

**Fig. 9:**
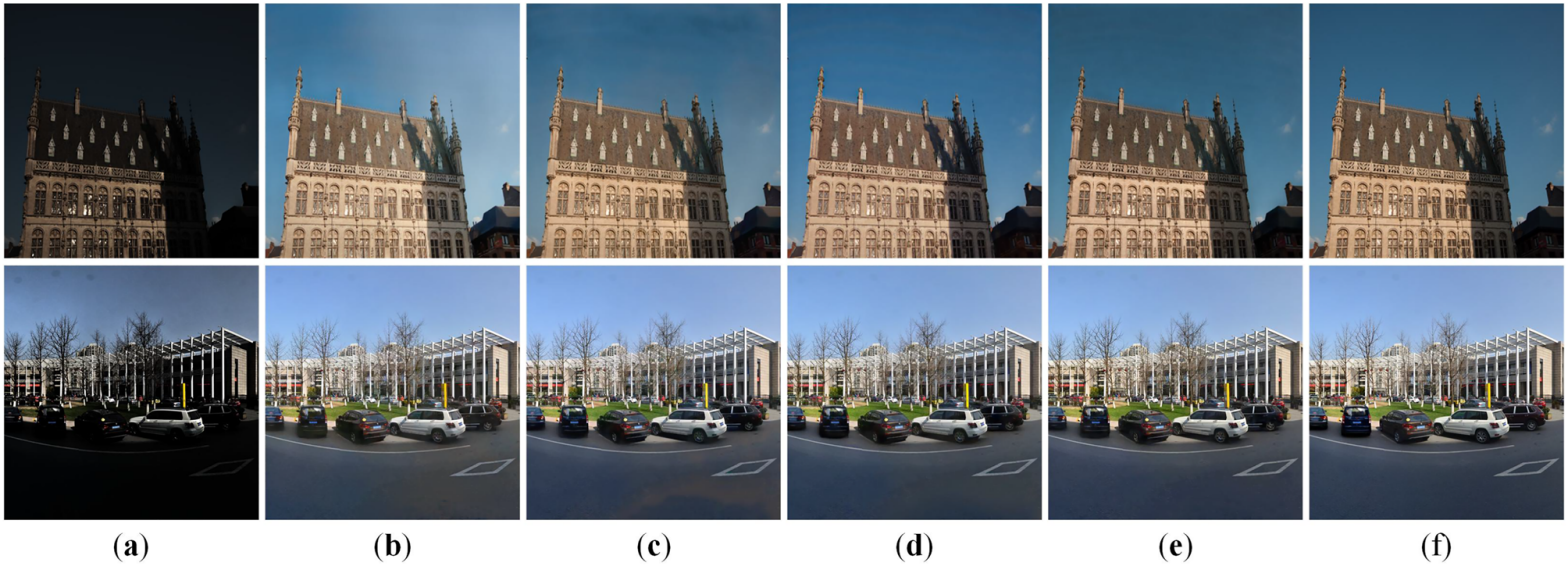
Visual ablation of SRU , ARFCA and SE. (**a**) Input. (**b**) w/o SRU. (**c**) w/o ARFCA. (**d**) Ours. (**e**) GT.

#### SRU

In our proposed network, images processed by the iterative enhancement strategy are treated as a pseudo-sequence, which is fed into the SRU. This allows the model to learn gradual brightness and detail enhancement, thereby improving low-light enhancement performance. To verify the efficacy of the SRU, we conducted ablation experiments comparing network performance with and without this module, assessing its impact on overall results.Objective results in Table 3 (across the three test datasets) show that removing the SRU leads to significant performance degradation: On LOL-v1, PSNR drops by 12.3% and LPIPS increases by 16.4%; on LOLv2-Synthetic, PSNR decreases by 10.9% and LPIPS surges by 32.7%; on TT100K-re, PSNR falls by 3.5% and LPIPS rises by 24.2%. These metrics indicate severe degradation in texture preservation and structural consistency when the module is excluded.

Visual comparisons in Fig. 9 further confirm this trend: without the SRU, the sky in the second row of images appears noticeably overexposed (whiter), differing significantly from normal-light images.

#### ARFCA

In our proposed network, each branch integrates an ARFCA module. This attention mechanism enhances the model’s perception of critical features by focusing on information-rich channels, while using fewer parameters and computational resources to improve efficiency in low-light image processing.

To investigate the impact of the ARFCA module, we conducted ablation experiments by comparing the full model with a variant lacking this module. Table 3 presents the objective results across the three test datasets, comparing performance with and without the ARFCA module. Removing the module reduces PSNR by 0.78% and increases LPIPS by 20.69% on LOL-v1; on LOLv2-Synthetic, PSNR drops by 3.69% and LPIPS rises by 10.91%; on TT100K-re, PSNR decreases by 2.93% and LPIPS increases by 21.21%. Visual comparisons in Fig. 9 further validate these findings. Notably, in the first row of images, removing the ARFCA module introduces uneven black patches on the wall, whereas the normally lit images and those enhanced by our method do not exhibit this issue.

Beyond validating its necessity, we further replaced the ARFCA with two widely used channel-attention modules–SE and ECA–under identical backbone and training settings. As shown in Table 4, ARFCA achieves the highest PSNR across all three datasets and the lowest MSE. The SSIM is comparable to or better than the alternatives; on TT100K-re, it is only 0.001 lower than the best baseline. LPIPS achieves the best performance on LOL-v1 and LOLv2-Synthetic, and the second-best on TT100K-re (0.033 vs. 0.031).

These results indicate that the adoption of the ARFCA module leads to better quantitative performance and visual quality, making it a key component for achieving high-quality low-light image enhancement in the overall network.

**Table 4:** Evaluation of PSNR/SSIM under alternative attention replacements for ARFCA.

	LOL-v1				LOLv2-Synthetic				TT100K-re			
Module	PSNR$\uparrow$	SSIM$\uparrow$	MSE$\downarrow$	LPIPS$\downarrow$	PSNR$\uparrow$	SSIM$\uparrow$	MSE$\downarrow$	LPIPS$\downarrow$	PSNR$\uparrow$	SSIM$\uparrow$	MSE$\downarrow$	LPIPS$\downarrow$
SE	23.08	0.827	85.242	0.130	24.61	0.911	77.902	0.071	32.81	0.956	20.992	**0.031**
ECA	23.04	0.829	85.555	0.145	24.80	0.924	77.017	0.066	32.99	**0.958**	21.482	0.036
**ARFCA(Ours)**	**23.33**	**0.829**	**83.627**	**0.116**	**25.48**	**0.931**	**72.879**	**0.055**	**33.50**	0.957	**19.452**	0.033

#### Iterative enhancement strategy

**Fig. 10:**
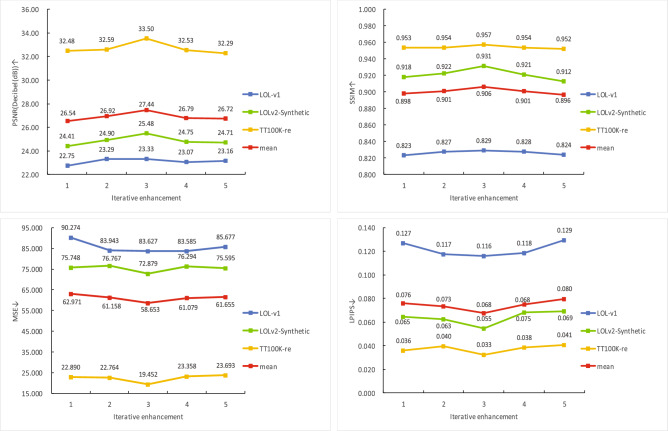
Evaluation of Average PSNR, SSIM, MSE and LPIPS at different iteration counts in three test datasets.

To assess the effectiveness of the iterative enhancement strategy and determine the optimal iteration count, we conducted ablation experiments by varying the number of iterations from 1 to 5. As illustrated in Fig. 10, the average PSNR and SSIM values across the three test datasets first increase and then decrease, while the MSE and LPIPS indicators first dropped and then rose. Both reached their optimal states after three iterations. All four metrics reach their optimal values after three iterations. At this optimal point, the model outperforms configurations with 1, 2, 4, and 5 iterations. Compared with the under-enhanced (iteration 1) and overfitted (iteration 5) states, iteration 3 achieves superior fidelity (e.g., +3.4% average PSNR vs. iteration 1) and lower perceptual error (e.g., ?15.0% average LPIPS vs. iteration 5). Intermediate iterations (2 and 4) exhibit moderate performance but fail to match iteration 3’s balance between accuracy and efficiency. While additional iterations may yield marginal metric improvements under controlled conditions, they also incur excessive computational costs and increase the risk of overfitting–ultimately compromising real-world generalization. Therefore, three iterations are adopted as the optimal trade-off among performance, computational efficiency, and robustness.

In addition, we performed an ablation on the concatenation strategy. The results in Table 5 indicate that show that double concatenation consistently outperforms no concatenation and single concatenation, validating that repeating the enhanced output once helps the network better focus on improved features without introducing redundant overhead.

**Table 5:** Evaluation of average PSNR, SSIM, MSE and LPIPS values for different concatenation strategies on three test datasets.

	LOL-v1				LOLv2-Synthetic				TT100K-re			
Module	PSNR$\uparrow$	SSIM$\uparrow$	MSE$\downarrow$	LPIPS$\downarrow$	PSNR$\uparrow$	SSIM$\uparrow$	MSE$\downarrow$	LPIPS$\downarrow$	PSNR$\uparrow$	SSIM$\uparrow$	MSE$\downarrow$	LPIPS$\downarrow$
No Concat	22.11	0.815	90.319	0.125	24.73	0.922	78.038	0.065	31.42	0.955	26.242	0.035
Single Concat	22.78	0.820	88.712	0.127	24.56	0.921	77.686	0.063	32.36	0.956	21.876	**0.033**
**Ours**	**23.33**	**0.829**	**83.627**	**0.116**	**25.48**	**0.931**	**72.879**	**0.055**	**33.50**	**0.957**	**19.452**	**0.033**

**Table 6:** Metrics of ablation experiments for different loss functions on three test datasets.

Model	LOL-v1				LOLv2-Synthetic				TT100K-re			
	PSNR$\uparrow$	SSIM$\uparrow$	MSE$\downarrow$	LPIPS$\downarrow$	PSNR$\uparrow$	SSIM$\uparrow$	MSE$\downarrow$	LPIPS$\downarrow$	PSNR$\uparrow$	SSIM$\uparrow$	MSE$\downarrow$	LPIPS$\downarrow$
w/o $\textrm{L}_{\textrm{Adv}}$	22.2	0.822	90.576	0.130	24.34	0.923	79.761	0.062	32.41	0.955	22.257	0.034
w/o $\textrm{L}_{\textrm{Color}}$	22.41	0.830	90.772	0.127	24.62	0.917	79.127	0.071	32.34	0.956	22.312	0.034
w/o $\textrm{L}_{\textrm{MP}}$	21.40	0.745	89.244	0.221	23.97	0.913	79.586	0.072	32.69	0.956	22.077	**0.032**
w/o $\textrm{L}_{\textrm{Pix}}$	22.88	**0.834**	91.267	0.123	25.02	0.920	76.930	0.061	32.74	0.956	21.854	**0.032**
**Ours**	**23.33**	0.829	**83.627**	**0.116**	**25.48**	**0.931**	**72.879**	**0.055**	**33.50**	**0.957**	**19.452**	0.033

#### Loss function

**Fig. 11:**
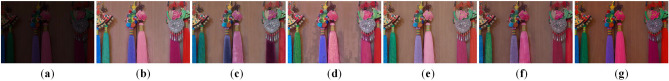
Loss-function ablation results. (**a**) Input. (**b**) w/o $L_{\textrm{Adv}}$. (**c**) w/o $L_{\textrm{color}}$. (**d**) w/o $L_{\textrm{MP}}$. (**e**) w/o $L_{\textrm{pix}}$. (**f**) Ours. (**g**) GT.

In our approach, four distinct loss functions synergistically optimize low-light image quality by addressing brightness, contrast, detail retention, and color restoration. To dissect their individual contributions, we conduct performance verification by removing each loss in turn while keeping the network architecture unchanged. Table 6 shows our full-loss model outperforms single-loss-ablation variants in 10 out of 12 metrics. Specifically: On LOL-v1, PSNR and MSE improve by 2.0% and 6.3% versus the second-best performance; on LOLv2-Synthetic, PSNR and LPIPS gain 1.8% and 11.3% over the suboptimal case; on TT100K-re, our method achieves a 2.5% improvement in PSNR, although LPIPS dose not reach the best score, the gap is only 0.001, indicating nearly equivalent perceptual quality. Furthermore, Fig. 11 provides a visual comparison of the ablation experiments on the loss function, where the absence of specific loss components leads to observable degradations such as reduced color saturation and patchy artifacts in the reconstructed images.

### Discussion and limitations

Although the proposed MBLLIE-Net achieves outstanding results on the LOL-v1, LOLv2-Synthetic, and TT100K-re datasets, certain challenges remain in specific scenarios.

**Fig. 12:**
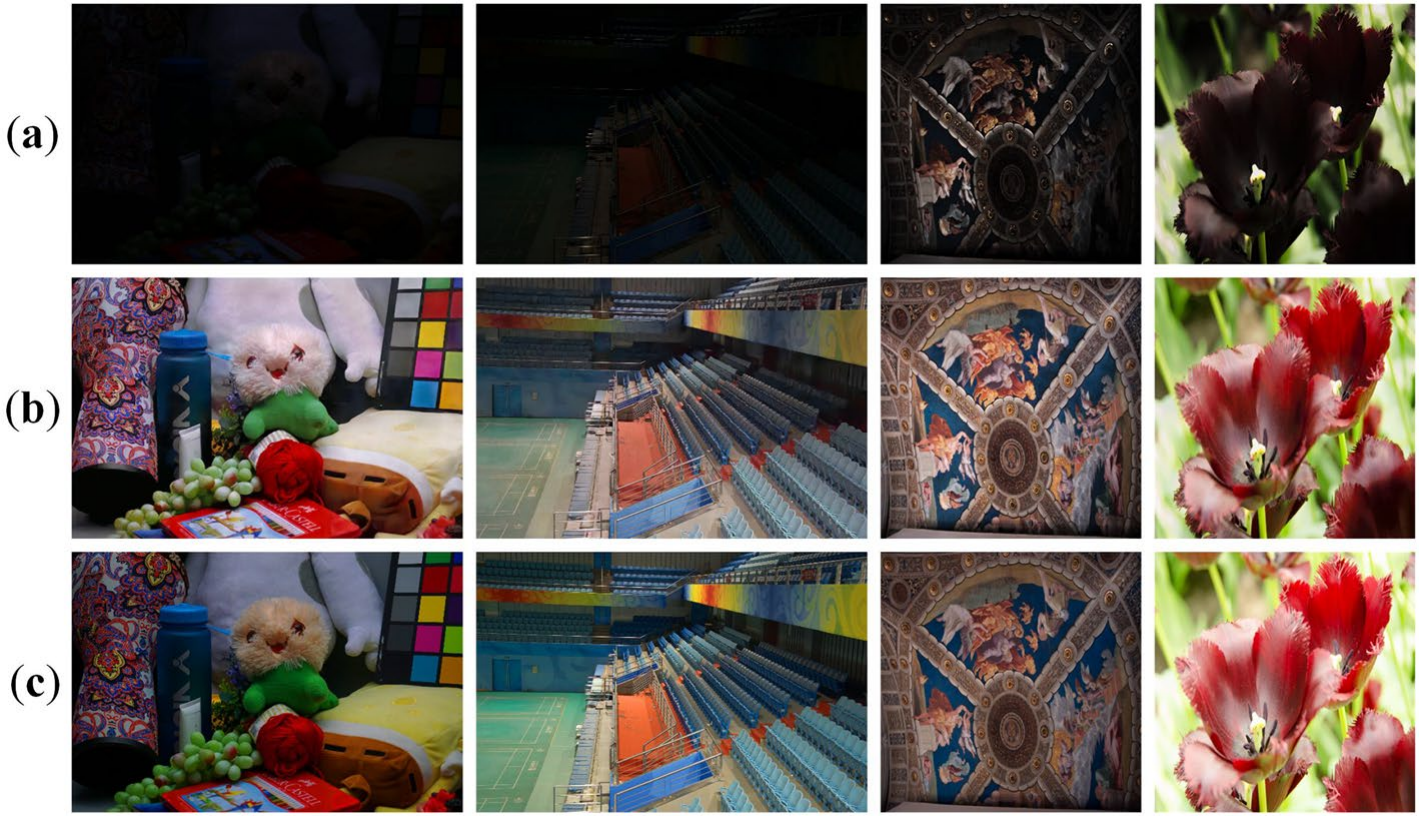
Failed cases of image enhancement. (**a**) Low light image. (**b**) MBLLIE-Net results. (**c**) GT.

First, under extreme illumination conditions–such as highly saturated regions or uneven lighting distributions–the model may produce slight color biases or over-brightened areas. As shown in Fig. 12, the yellow toy in the lower-right corner of the first-row image appears whitened after enhancement, while the painting region in the third-row image exhibits excessive brightness compared with the ground truth. This phenomenon suggests that the current design still has limitations in local color adaptation. Despite employing an optimization strategy that combines multiple loss functions, the enhanced images may still display localized color distortions. These issues could potentially be mitigated by improving data augmentation strategies under extreme illumination, enhancing feature extraction capability, and introducing more fine-grained local scene modeling, thereby further improving the visual consistency and robustness of the results.

Second, the model is trained on paired datasets containing both synthetic and real low-light images. While it demonstrates good generalization across multiple datasets, its performance may degrade when applied to domains with significantly different noise distributions or unseen illumination conditions. Future work could incorporate unsupervised or domain-adaptive learning strategies to further enhance robustness.

In addition, the proposed multi-branch architecture inevitably introduces certain computational overhead. Future research could explore more efficient architectural designs and parameter compression techniques to enable real-time deployment on mobile or embedded devices while maintaining enhancement quality.

## Conclusion

This paper proposes a Multi-Branch Low-Light Image Iterative Enhancement Network (MBLLIE-Net) for low-light image enhancement. The network adopts a U-Net-like architecture with four multi-scale enhancement branches, where each branch integrates an ARFCA attention module and an SRU module to extract and refine scale-specific features, thereby enabling adaptation to diverse low-light conditions. An iterative enhancement strategy is employed to progressively refine preliminary results, producing finer and more accurate outputs. In addition, the network is optimized using a weighted combination of four loss functions–adversarial, pixel, color, and multi-scale losses–which further improve overall image quality. Experimental results on three benchmark datasets and three real-world datasets demonstrate that MBLLIE-Net effectively enhances images under various low-light conditions, generating outputs with natural colors and clear textures.

These findings not only validate the potential of the proposed framework for robust low-light image enhancement in complex real-world environments but also highlight its practical relevance. In particular, reliable low-light enhancement is of great importance for applications such as traffic scene understanding, video surveillance, and autonomous driving, where visibility under adverse illumination is crucial. Looking ahead, we plan to further explore strategies to enhance generalization and computational efficiency, thereby extending the applicability of MBLLIE-Net to a broader range of scenarios. We believe that the concepts and methods presented in this study will provide new insights for the development of efficient, robust, and perceptually consistent image enhancement systems.

## Data Availability

The datasets generated and/or analysed during the current study are available in the MBLLIE-Net repository, https://github.com/Autumn1t/MBLLIE-Net.
